# The effect of age on the risk of early cardiac implantable electronic device infection

**DOI:** 10.1016/j.hroo.2025.04.001

**Published:** 2025-04-09

**Authors:** Meeri Honkanen, Hanna Viskari, Essi Ryödi

**Affiliations:** 1 Department of Internal Medicine, Tampere University Hospital, Tampere, Finland; 2 Faculty of Medicine and Health Technology, Tampere University, Tampere, Finland; 3 Heart Hospital, Tampere University Hospital, Tampere, Finland

**Keywords:** Age, Pacemaker infection, Risk factors, Healthcare-associated infection, Epidemiology

**Key Findings**

▪ The results of previous studies examining the effect of age on risk of cardiac implantable electronic device infection have been conflicting. In the current study, age was not a risk factor for early cardiac implantable electronic device infection.
▪ Previous studies have not assessed the effect of age specifically for early cardiac implantable electronic device infections.
▪ Younger age is included in some cardiac implantable electronic device infection risk-prediction models. When assessing these models, the timing of infection should be taken into account.

Risk factors for cardiac implantable electronic device (CIED) infections are well studied. However, studies assessing the risk factors for early infections occurring within 3 months from the latest procedure are lacking. Furthermore, the results of studies examining the effect of age on risk of CIED infection have been conflicting.^1^ The aim of this study was to examine the effect of age on risk of early CIED infection.

The study included patients with CIED implantations or modification procedures performed at the Heart Hospital, Tampere University Hospital, between 2013 and 2021 (n = 7906 patients). CIED infections occurring within 90 days from the procedure were identified from the Heart Hospital’s prospectively collected electronic database (reported infections and CIED removals because of infection) (Kardio Heart Database, Finland) and from the health care-associated infection register of the Tampere University Hospital (SAI, Neotide Corporation, Vaasa, Finland). Patients without CIED infection who died within 90 days from the procedure were excluded (n = 168). Data on potential risk factors for infection were collected from the Kardio database. Intravenous antibiotic prophylaxis with cefuroxime is routinely given to patients 1 hour before incision, as per hospital protocol. The research reported in this paper was conducted along the Helsinki Declaration and adhered to the Strengthening the Reporting of Observational Studies in Epidemiology (STROBE) guidelines for reporting of observational studies. Permission to conduct this study was granted by the research director of the Research Services of the Pirkanmaa Hospital District. As the study was registry based, informed patient consent was waived according to national legislation.

All data analyses and management were performed using the SPSS for Windows 29.0 statistical software package (IBM, Chicago, IL). Categorical variables were compared with χ^2^ test or Fisher’s exact test and continuous variables with Student independent-samples *t* test. A value of *P* < .05 was considered statistically significant. The association of age on the risk for CIED infection was analyzed using binary logistic regression with univariate analysis; odds ratios (ORs) and 95% confidence intervals (CIs) were calculated. In addition, a multivariable model including age and all variables associated with the dependent variable (body mass index [BMI] and type of procedure) on bivariable analysis (*P* < .1) was developed. Age and BMI were analyzed as continuous variables.

The characteristics of the study population are shown in Table 1. There were overall 8524 CIED procedures with 35 early CIED infections during the follow-up period. Of the procedures, 71% were primary CIED placements and the remaining 29% modification or generator-replacement procedures. The incidence of early CIED infection was 0.4%. The median time between the procedure and the occurrence of infection was 22 days (range 3–77 days), and 23 of the infections (66%) occurred within 1 month.

**Table 1:** Description of the study population with and without early CIED infections and the results of multivariable logistic regression analysis

	Procedures with CIED infection (n = 35)		Procedures without CIED infection (n = 8489)		Total (n = 8524)		*P* value	OR	95% CI
	n	%	n	%	n	%			
Age (years, mean, SD)	70 (SD ± 16)		74 (SD ± 13)		74 (SD ± 13)		.09	0.98	0.95–1.02
Male gender	18	51	4761	56	4779	56	.58		
BMI (kg/m^2^, mean, SD)∗	30 (SD ± 6)		28 (SD ± 5)		28 (SD ± 5)		.06	1.06	0.99–1.14
Diabetes†	7	39	1258	28	1265	28	.29		
Hypertension‡	11	85	2455	67	2466	68	.19		
Primary CIED placement	30	86	6013	71	6043	71	.053	0.70	0.20–2.41
Indication for CIED							.79		
AV block	14	40	2661	31	2675	31			
SSS	11	31	2373	28	2384	28			
Chronic AF	5	14	1351	16	1356	16			
Cardiomyopathy	2	6	1178	14	1180	14			
Ventricular arrhythmia	2	6	630	7	632	7			
Chronic heart failure	1	3	230	3	231	3			
Other	0	0	66	1	66	1			
Type of CIED							.62		
PPM	29	83	6305	74	6334	74			
ICD	4	11	1101	13	1105	13			
CRT	2	6	1044	12	1046	12			
Micra	0	0	39	0.5	39	0.5			
The urgency of the procedure							.84		
Urgent	16	46	3733	44	3748	44			
Elective	19	54	4756	56	4775	56			
Temporary pacemaker before procedure	1	3	950	11	951	11	.12		

AF = atrial fibrillation; AV = atrioventricular; BMI = body mass index; CI = confidence interval; CIED = cardiac implantable electronic device; CRT = cardiac resynchronization therapy; ICD = implantable cardioverter defibrillator; OR = odds ratio; PPM = permanent pacemaker; SD = standard deviation; SSS = sick sinus syndrome.

∗ Information available for 3747 (44%) patients (19 CIED infections and 3728 without infections).

† Information available for 4570 (54%) patients (18 CIED infections and 4552 without infections).

‡ Information available for 3653 (43%) patients (13 CIED infections and 3640 without infections).

Despite the large number of procedures included in this study, no specific risk factor for an early CIED infection was identified (Table 1). Age was not associated with an increased risk of CIED infection in the univariate (OR 0.98, 95% CI 0.96–1.00) or multivariable (OR 0.98, 95% CI 0.95–1.02) analysis, Also not associated with an increased risk for infection were gender, indication for CIED, type of CIED, urgency of the procedure, or use of temporary pacemaker before the procedure (Table 1).

Age was not associated in this large register-based study with a risk of developing a CIED infection. The results of previous studies have been conflicting. In a large meta-analysis, Polyzos et al^2^ did not find an association between age and risk of CIED infection. In a large nationwide registry study, however, Olsen et al^3^ showed that younger age was associated with an increased risk of CIED infection. Younger age has been included in some CIED infection risk-prediction models as well.^4^

Previous studies have not assessed the effect of age specifically for early CIED infections. In their study, Sohail et al^5^ examined the risk factors for implantable cardioverter-defibrillator device infection separately for infections occurring before and after 6 months from the procedure, but their study did not assess the effect of age, as the controls were matched for age. However, their study showed that the risk factors for infection differ between early- and late-onset CIED infections.

There are limitations to the current study that must be acknowledged. Because of the retrospective nature of the study, data on many patient-related risk factors was not available, and thus it was not possible, for example, to assess the effect of anticoagulation, immunosuppression, kidney function, or malignancy on the risk of CIED infection. Also, even though this is the largest study assessing the effect of risk factors for early CIED infection, it would have required more than 50,000 procedures to have adequate power, which is not feasible in a single-center study. In addition, the number of infections was so low that it is difficult to identify infection predictors. Furthermore, the majority of CIEDs were permanent pacemakers (PPMs); even though in the current study there was no difference in the incidence of infection among different types of CIEDs, PPMs generally have a lower risk for infection.

The results of this study suggest that age is not a significant risk factor for early CIED infections. When assessing the different CIED infection risk-prediction models, the timing of infection should be taken into account. This should be acknowledged in future validation studies of these models as well.

## References

[1] Blomström-Lundqvist C., Traykov V., Erba P.A. (2020). European Heart Rhythm Association (EHRA) international consensus document on how to prevent, diagnose, and treat cardiac implantable electronic device infections. EP Europace.

[2] Polyzos K.A., Konstantelias A.A., Falagas M.E. (2015). Risk factors for cardiac implantable electronic device infection: a systematic review and meta-analysis. Europace.

[3] Olsen T., Jørgensen O.D., Nielsen J.C. (2022). Risk factors for cardiac implantable electronic device infections: a nationwide Danish study. Eur Heart J.

[4] Birnie D.H., Wang J., Alings M. (2019). risk factors for infections involving cardiac implanted electronic devices. J Am Coll Cardiol.

[5] Sohail M.R., Hussain S., Le K.Y. (2011). Risk factors associated with early- versus late-onset implantable cardioverter-defibrillator infections. J Interv Card Electrophysiol.

